# Balancing ideals and realities: health care professionals’ perspectives of and attitudes toward digital patient-centered cancer care

**DOI:** 10.1007/s11136-025-04103-w

**Published:** 2026-02-18

**Authors:** Elias David Lundereng, Alen Brkic, Kate Absolom, Elisabeth Andvik, Kim Beernaert, Kathrin Cresswell, Olav Faisal Dajani, Nienke De Glas, Marie Fallon, Victoria Freitas-Durks, Kristin Vassbotn Guldhav, Marianne Jensen Hjermstad, Stein Kaasa, Geana Paula Kurita, Jo-Åsmund Lund, Nicoleta Mitrea, Steven Olde Damink, Ørnulf Paulsen, Guro Meldre Pedersen, Terese Solvoll Skåre, Tonje Lundeby, The MyPath consortium, Kate Absolom, Morten Andresen, Marek Atter, Dag Ausen, Sara Bea, Kim Beernaert, Augusto Caraceni, Andres Cervantes, Kathrin Cresswell, Olav Dajani, Judith de Vos-Geelen, Luc Deliens, Felicity Evans, Marie Fallon, Victoria Freitas Durks, Teresa Serra Cassano, Inez Gonzalez-Barrallo, Peter Hall, Marianne Jensen Hjermstad, Marisol Huerta, Kristin Solheim Hustad, An Jacobs, Stein Kaasa, Lisa Heide Koteng, Geana Paula Kurita, Henrik Larsen, Ulrik Lassen, Nicola Jane Latino, Tonje Lundeby, Camilla Charlotte Lykke, Giacomo Massa, Ulla Mathiesen, Nicoleta Mitrea, Daniela Mosoiu, Steven Olde Damink, Helle Pappot, Koen Pardon, Cathy Payne, Oana Predoiu, Anne-Lore Scherrens, Morena Shkodra, Per Sjøgren, Eivind Storaas, Amaia Urrizola, Peder Heyderdahl Utne, Femke Van Landschoot, Galina Velikova, Lorraine Warrington, Naomi White, Robin Williams, Adam Stuart Dowrick, Ragnhild Lund Schultz, Julia Götz, Patrick Schneier, Jeanette Müller, Julien Luyten, Anouk de Wilde

**Affiliations:** 1 European Palliative Care Research Centre (PRC), Department of Oncology, Oslo University Hospital, and Institute of Clinical Medicine, University of Oslo, Oslo, Norway; 2 Leeds Institute of Health Services Research, Leeds Institute of Medical Research, University of Leeds, Leeds, UK; 3 BI Norwegian Business School, Bergen, Norway; 4 End-of-Life Care Research Group, Vrije Universiteit Brussel (VUB) & Ghent University, Brussels, Belgium; 5 Usher Institute, The University of Edinburgh, College of Medicine and Veterinary Medicine, University of Edinburgh, Edinburgh, UK; 6 Department of Medical Oncology, Helse Førde, Svanehaugvegen 2, 6812 Førde, Norway; 7 INCLIVA Instituto de Investigación Sanitaria, Valencia, Spain; 8 MATRIX, Norwegian Centre for Clinical Cancer Research, Oslo, Norway; 9 Department of Clinical Medicine, Faculty of Health and Medical Sciences, University of Copenhagen, Copenhagen, Denmark; 10 Pain and Palliative Care Research Group, Multidisciplinary Pain Centre at Department of Anaesthesiology, Pain and Respiratory Support, Section of Palliative Medicine at Department of Oncology, Rigshospitalet, Copenhagen University Hospital, Copenhagen, Denmark; 11 Department of Health Sciences Ålesund, Faculty of Medicine and Health Sciences, Norwegian University of Science and Technology, Ålesund, Norway; 12 Clinic for Cancer Treatment and Rehabilitation, Møre and Romsdal Health Trust, Ålesund, Norway; 13 Department of Fundamental Disciplines and Clinical Prevention, Faculty of Medicine, University of Transilvania, Brasov, Romania; 14 Department of Education and Research, HOSPICE Casa Sperantei, Brasov, Romania; 16 Department of Oncology and Hematology, Telemark Hospital Trust, 3710 Skien, Norway; 17 DNV AS, Oslo, Norway; 18 Leeds Cancer Centre, St James’s University Hospital, Leeds, UK; 19 Leeds Institute of Medical Research at St James’s, University of Leeds, Leeds, UK; 20 DNV Imatis AS, Porsgrunn, Norway; 21 Institute of Genetics and Cancer, University of Edinburgh, Edinburgh, UK; 22 Department of General Practice and Chronic Care, End-of-Life Care Research Group, Vrije Universiteit Brussel (VUB) & Ghent University, Brussels, Belgium; 23 Fondazione IRCCS Istituto Nazionale Dei Tumori, Milan, Italy; 24 Dipartimento di Scienze Cliniche e di Comunità – Dipartimento di eccellenza 2023 – 2027 , Università Degli Studi Di Milano, Milan, Italy; 25 Department of Medical Oncology INCLIVA, Biomedical Research Institute, University of Valencia, Valencia, Spain; 26 CIBERONC, Instituto Salud Carlos III, Madrid, Spain; 27 Usher Institute, University of Edinburgh, Edinburgh, UK; 28 Department of Internal Medicine, Division of Medical Oncology, GROW - Research Institute for Oncology & Reproduction, Maastricht University Medical Center+, Maastricht, The Netherlands; 29 Department of Scientific & Medical Affairs, European Society for Medical Oncology (ESMO), Lugano, Switzerland; 30 Department of Medical Oncology INCLIVA, Biomedical Research Institute, Valencia, Spain; 31 Department of Media and Communication Studies, Imec-SMIT Research Group, Vrije Universiteit Brussel (VUB), Brussels, Belgium; 32 Department of Oncology and Department of Anaesthesiology, Pain and Respiratory Support, Rigshospitalet Copenhagen University Hospital, Copenhaguen, Denmark; 33 Department of Clinical Medicine, University of Copenhagen, Copenhaguen, Denmark; 34 Department of Oncology, Section of Palliative Medicine, Rigshospitalet Copenhagen University Hospital, Copenhaguen, Denmark; 35 Department of Oncology, Rigshospitalet Copenhagen University Hospital, Copenhaguen, Denmark; 36 Department of Oncology and Palliative Care, North Zealand Hospital, North Zealand, Denmark; 37 Department of Fundamental Disciplines and Clinical Prevention, Faculty of Medicine, University of Transilvania From Brasov, Brasov, Romania; 38 Department of Education and National Development, HOSPICE Casa Sperantei, Brasov, Romania; 39 Department of Medical and Surgical Specialties, Faculty of Medicine, University of Transilvania From Brasov, Brasov, Romania; 40 Department of Surgery, Maastricht University Medical Centre, Maastricht, The Netherlands; 41 NUTRIM Institute of Nutrition and Translational Research in Metabolism, Maastricht University, Maastricht, The Netherlands; 42 Department of General, Visceral, Vascular and Transplant Surgery, University Hospital Essen, Essen, Germany; 43 European Association for Palliative Care, Vilvoorde, Belgium; 44 Institute for the Study of Science, Technology and Innovation, University of Edinburgh, Edinburgh, UK

**Keywords:** Patient-centered care, PROMs, Oncology, Quality of life, Digital health, Implementation science, Qualitative research

## Abstract

**Purpose:**

Patient-centered care (PCC) improves quality of life, symptom management and healthcare outcomes in oncology. However, integration into routine cancer care remains limited. Digital solutions using patient-reported outcome measures (PROMs) offer a potential mechanism to operationalize PCC. This study explored healthcare professionals’ (HCPs) pre-implementation perspectives on using digital PROMs to support PCC in Norwegian oncology outpatient clinics, informing the design and implementation strategies of the European MyPath digital solution.

**Methods:**

Semi-structured interviews (n = 29) and three focus groups (n = 16) were conducted with varied HCPs across four Norwegian hospitals. Interviews explored perceptions of PCC, experiences with PROMs, and requirements for digital implementation. Data were analyzed using thematic analysis, combining inductive and deductive coding guided by the TPOM framework.

**Results:**

Four themes emerged: (1) balancing PCC with disease-centered practices, (2) integrating PCC into daily routines, (3) customization and patient acceptance of digital tools, and (4) combining patient-reported data with clinical autonomy. HCPs viewed digital PROMs as promising for facilitating PCC but emphasized that successful implementation requires workflow alignment, adaptable digital solutions, and strong stakeholder engagement. Concerns included patient digital literacy, workload implications, and overreliance on PROMs at the expense of direct patient interaction.

**Conclusion:**

Our findings highlight a tension between HCPs’ needs for technical functionality and workflow alignment, and the support required to adapt their practice to fully realize PCC through digital tools. Integrating PCC successfully requires organizational, cultural, and workflow adaptations, alongside active HCP engagement in design and implementation. These changes are essential to reposition PCC as an integral rather than competing component of high-quality cancer care.

**Supplementary Information:**

The online version contains supplementary material available at 10.1007/s11136-025-04103-w.

## Abstract (plain language summary)

Cancer treatment often focuses primarily on treating the tumor, while the emotional, physical, and psychological impacts on patients are overlooked. These factors can significantly affect patients' quality of life during and after treatment. The study addresses the challenge of integrating patient-centered care into cancer treatment, an approach that considers patients' overall well-being, not just their cancer. This study is part of the European Union-funded project MyPath, that aims to develop a digital tool supporting more patient-centered cancer care in Europe. Understanding the perspectives of healthcare providers is essential in ensuring that the tool fits with real-world clinical settings. The main point of this study is to explore how healthcare providers view patient-centered care and the role of digital tools in improving quality of life through better symptom management. The results show that healthcare providers recognize the importance of enhancing quality of life and believe that digital tools could support a more patient-centered approach to care. However, they tend to view patient-centered care as more relevant to end-of-life or palliative care, rather than active cancer treatment. This suggests that while digital tools hold potential to improve quality of life, a shift in mindset and hospital cultures is needed to make patient-centered care an equal part of cancer treatment, rather than a competing focus.

**Supplementary Information:**

The online version contains supplementary material available at 10.1007/s11136-025-04103-w.

## Introduction

Patient-centered care (PCC) is a healthcare model that prioritizes patients’ quality of life (QoL) by being “respectful of and responsive to individual patient preferences, needs, and values, and ensuring that patient values guide all clinical decisions” [1]. Integrating PCC into routine cancer care has been shown to improve symptom management, patient satisfaction, QoL, healthcare efficiency, and even survival [2–7]. This evidence has prompted major initiatives, including the European Union’s Mission on Cancer emphasizing QoL and PCC, and organizations such as the World Health Organization (WHO), the European Society for Medical Oncology (ESMO), and the American Society for Clinical Oncology (ASCO) advocating for systematic incorporation of PCC across oncology, regardless of tumor type or treatment intent [4, 5, 8–12].

Despite these efforts, oncology care remains largely disease-centered, with a predominant focus on treating the disease. Systematic integration of PCC is challenged by political, societal, organizational, and professional barriers, including emphasis on curative treatment, limited resources, lack of standardized frameworks, and time constraints or insufficient training in shared decision-making [13–22]. Implementation is further complicated by abstract definitions of PCC, which often lack tangible clinical relevance for healthcare providers (HCPs) [23–26].

Patient-reported outcome measures (PROMs) have emerged as key tools for operationalizing PCC. PROMs provide a structured way to capture patient experiences, offering HCPs a holistic view of patients’ conditions and facilitating more patient-centered consultations [27, 28]. Digital PROMs, enabled through health information technologies (HIT), offer opportunities to integrate PROMs into clinical workflows, moving beyond cumbersome paper-based formats [29–35]. However, many digital PROM systems remain confined to research settings, lack strategies for scalable, real-world implementation, or focus on selected phases of the cancer trajectory or specific tumor types [30, 35, 36]. Consequently, despite robust evidence of benefit, digital PROMs are inconsistently integrated in routine oncology practice.

The MyPath project is an EU-funded innovation and implementation science project that seeks to develop and implement a comprehensive digital solution to support PCC in cancer care. Conceptually, the MyPath digital solution aims to integrate digital PROMs with evidence-based guidance for symptom management and follow-up, enabling HCPs to access patient-reported outcomes and use them systematically with patients to facilitate shared decision-making, making consultations more patient-centered [37–44]. Implementing MyPath will require a shift in clinical practice, introducing both new tools and priorities. HCPs play a critical role as gatekeepers of change, making their perspectives essential for successful adoption [23, 30]

Although digital tools and PROMs have been studied in oncology, most research has focused on primary or palliative care settings or selected patient populations [31, 44–47]. Evidence is limited regarding HCPs’ experiences with digitally supported PCC using PROMs across oncology specialties and phases of care, including treatment, survivorship, and palliative care. Understanding these perspectives is essential for informing the design and implementation of innovative solutions like MyPath, ensuring clinically relevant, useful and sustainable digital solutions that are scalable across the entire cancer care continuum.

The primary aim of this study was to explore HCPs’ perspectives on digitally supported PCC using PROMs through the proposed MyPath digital solution in oncology outpatient clinics. The study sought to understand: 1) How PCC is currently conceptualized and practiced in routine oncology care today; 2) HCPs experiences using PROMs to support clinical consultations; and 3) HCPs perceived requirements for design and functionality to digitally supported PCC.

## Methods

MyPath, an EU-funded project, aims to implement a digitally enabled PCC approach across nine European cancer centers using a theory-based, mixed-methods implementation science approach that combines formative and summative evaluations to iteratively inform and assess development and implementation in real-world settings [48]. The Technology, People, Organizations, and Macroenvironment (TPOM) framework is the evaluative framework, chosen for its evidence-based assessment of factors influencing HIT implementation [49].

Central to MyPath’s strategy is continuous stakeholder engagement throughout its three phases: development, implementation, and evaluation. This study reports on the formative, developmental phase of MyPath, presenting findings from qualitative interviews with HCPs in the Norwegian study arm. Insights from this study intend to inform the design and implementation strategies of MyPath, ensuring that the solution aligns with real-world oncology practice and supports a shift to PCC in oncology [50].

### Design

This study employed qualitative, interpretive and descriptive design, grounded in Interpretive Description (ID), chosen for its suitability in generating practical, contextually relevant knowledge to guide development and implementation strategies [51]. The objective was to understand interdisciplinary HCPs’ perspectives and experiences regarding PCC and PROMs across oncology outpatient clinics in Norway.

### Participants and recruitment

A total of 45 participants were recruited from four Norwegian hospitals between 2022–2023. The participants were selected through a purposive sampling strategy, guided by a stakeholder analysis conducted by the MyPath research team and local principal investigators (PIs). Snowball sampling was used to identify additional participants. We aimed for a diverse sample to ensure comprehensive representation across a range of treatment modalities and professional roles. The participants included a combination of physicians, nurses, and allied health professionals with experience in various cancer types and treatment modalities, including cancer survivorship, radiation treatment, surgical oncology, medical oncology, and palliative care. These groups were selected to ensure a broad representation of the entire cancer care continuum.

Individual interviews took place at a large urban university hospital, while three focus groups were conducted at hospitals in smaller, more rural parts of Norway. Due to the study's formative nature, data saturation was not pursued or assessed. Instead, all identified participants from the stakeholder analysis were approached to ensure comprehensive representation. The participant characteristics are provided in Table 1.

**Table 1:** Participant characteristics (n = 45)

Characteristic	Individual interviews (n = 29)	Focus group interviews 3 groups (n = 16)
**Sex**		
Female	21	11
Male	8	5
**Age**		
Years, mean	49·1	45·8
Median (range)	48 (31–63)	52 (29–57)
**Diagnostic group**		
Pancreatic cancer	15	
Head and neck cancer	3	
Testicular cancer	7	
Palliative care	4	
General oncology		16
**Professional background**		
Physician, oncologist or surgeon	13	8
Nurses with various specialties	12	8
Allied health professional (social worker, nutritionist, psychologist or secretary)	4	
**Experience**		
Working with cancer patients (years), mean	17·1	16
Median (range)	19 (1–38)	12·5 (1–38)

### Data collection

Data were collected through 29 semi-structured individual interviews and three focus groups with a total of 16 participants. Each participant was interviewed once, and interviews were conducted at the participants' workplaces, audio-recorded, and transcribed verbatim. The interviews were led by authors EDL, AB and TL, including two master’s students under EA’s supervision. The interviews were guided by a semi-structured guide developed collaboratively within MyPath, covering three key areas: (1) current management of patients’ physical, emotional, and social concerns; (2) experiences using PROMs in clinical practice; and (3) perspectives on how digital tools could support PCC. Prior to the interviews, participants received a standardized presentation outlining the project aims and conceptual aspects of the MyPath solution, including illustrative flowcharts. The interview guide is provided in Box 1.

**Box 1** Sample interview guide

**Figure:**
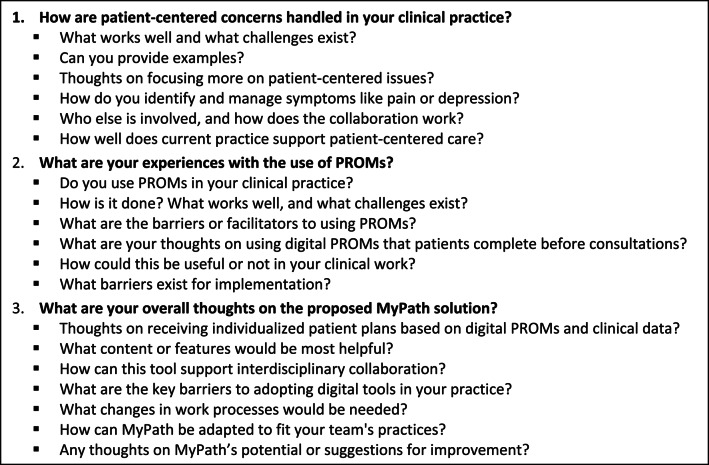

### Data analysis

**Fig. 1:**
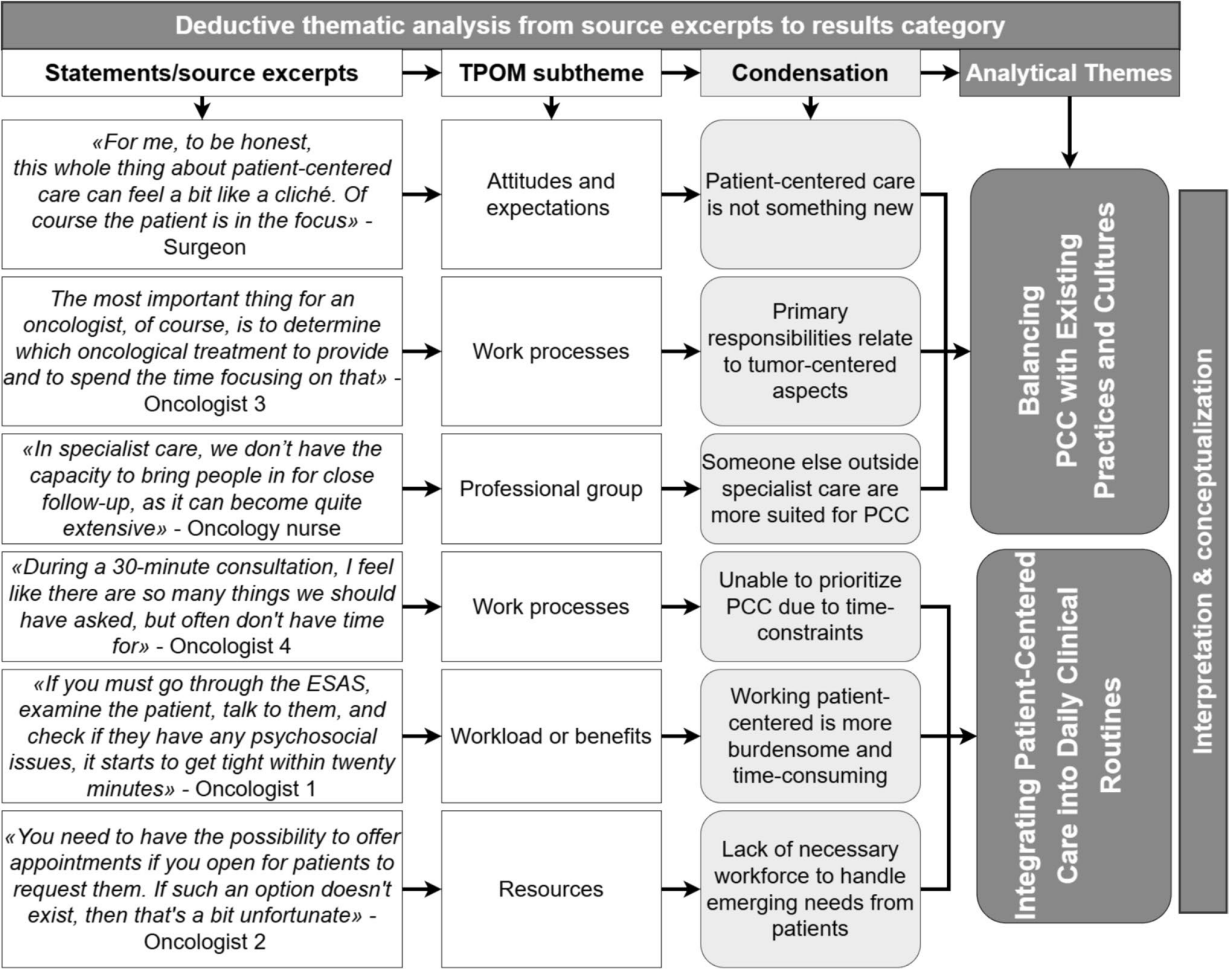
Example of the analysis process from source excerpts to two result categories

The data were analyzed using thematic analysis as outlined by Braun and Clarke [52], combining an inductive and deductive approach guided by the TPOM framework. Transcripts were read line-by-line by the first author (EDL) multiple times to achieve immersion, followed by a deductive coding guided by the TPOM framework, with simultaneous inductive coding to capture emergent concepts not covered by the framework. Codes were reviewed subtheme by subtheme and condensed into descriptions that captured the main essence of each subtheme. All subtheme condensations were reviewed by the first and last authors, and patterns were identified across main themes and subthemes, synthesizing and abstracting the data to develop credible descriptions grounded in the participants voices that addressed the study objectives. NVivo (QSR International) was utilized to organize, store, and facilitate the analysis of data. Examples of the analysis process are presented in Fig. 1.

### Rigor and trustworthiness

Several strategies were employed to ensure the rigor and trustworthiness of the findings. Credibility was strengthened through a systematic data analysis process, including regular meetings among researchers from different MyPath sites to discuss emerging findings and resolve interpretive differences. The research team represented diverse academic (social and medical sciences) and professional backgrounds, fostering rich reflexive discussions.

The present analysis was led by a PhD candidate with a background in oncology nursing and palliative care, supervised by senior researchers with expertise in health services research, digital health, oncology, palliative care, change management and behavioral psychology. Recognizing that prior experience with digital innovations, palliative care and management could influence interpretations, the team engaged in ongoing reflexive dialogue to examine how perspectives may have shaped the research questions, interview process, and data analysis.

Additionally, preliminary findings were presented in two separate meetings to a subset of local PIs and interview participants allowing for member checking and validation of the findings, further reinforcing credibility. Dependability was supported through detailed documentation of coding and theme development, facilitated by NVivo software. The study adhered to COREQ guidelines for qualitative research reporting [53] (File S1).

### Results

Four themes were identified through the analysis. 1) *Balancing Patient-Centered Care with Existing Hospital Practices and Cultures* - where participants described how the prioritization of anticancer treatment often sidelined PCC. 2) *Integrating Patient-Centered Care into Daily Clinical Routines* - in which the lack of standardized tools was seen as limiting the consistency of providing PCC. 3) *Customization Needs and Patient Acceptance of Digital Tools* - where participants emphasized the importance of tailoring a tool like MyPath to the specific patient groups they worked with. And 4) *Combining Patient-Reported Data with Clinical Autonomy* - where participants highlighted that patient-reported information should support rather than replace clinical judgment in PCC.

### Balancing patient-centered care with existing hospital practices and cultures

This theme highlights how oncology’s disease-centered culture shaped participants’ views on PCC. Most participants described a culture where anticancer treatment was prioritized, with symptom assessment often viewed as secondary or more relevant in palliative care settings. Participants reflected on the culture of oncology practice, their professional responsibilities, and the optimal setting for PCC in oncology.

Many clinicians recognized the importance of PCC, however they often viewed it as most relevant in palliative care settings, where symptom management and QoL were prioritized. In curative care or in settings where life-prolonging treatment was administered, patients were often seen as either not significantly burdened by symptoms or eligible for treatment regardless of symptom load, limiting the perceived usefulness and relevance of a solution like MyPath. As one oncology nurse explained:

*“A lot is done to ensure they get cured. It takes a lot to withhold treatment because of symptoms. Of course, we consider them, but our focus is on providing curative treatment and saving lives regardless.”* – Oncology nurse

This curative focus, where PCC was sidelined over anti-cancer treatment, was especially prominent among those working with patients with favorable prognosis or where curation was likely. In this setting PCC was often seen as an additional burden that could and should be managed by other less specialized HCPs. One oncologist emphasized this responsibility clearly:

*“My responsibility is the cancer treatment... That is the most important responsibility I have. After all, I’m not their general practitioner.”* – Oncologist

However, participants working with advanced cancers, such as pancreatic cancer, were more likely to see the value of integrating symptom management and PCC into routine practice:

*“I monitor the patients closely and make a plan from A to Z and try to assess symptoms in my regular consultations or refer them to the palliative care team if needed.”* – Oncologist

Across groups participants also highlighted that they viewed primary care services (e.g. general practitioners, home care services) as essential professionals in handling PCC, as the patients they treated spent most of their time at home. They worried that a focus on symptoms and emotional distress in hospitals would shift care responsibilities from the primary care services to the hospitals, undermining efforts to achieve the opposite, as illustrated by an oncologist:

*“A self-reported system might make patients rely too heavily on the hospital...we must ensure continuity of care at home.”* – Oncologist

### Integrating patient-centered care into daily clinical routine

This theme describes how adopting a PCC approach was challenged by existing workflow structures. While anticancer treatment adhered to standardized protocols, PCC depended on individual judgment and experience. The lack of standardized procedures to deliver PCC led to inconsistent practices, with symptom inquiries often limited to anticancer treatment-related issues. Participants viewed digital tools like MyPath as potentially helpful in providing structure to symptom assessment and follow-up. One oncologist reflected on the lack of standardized approaches to PCC and symptom management leading to inconsistency:

*“It’s not standardized. We ask about neuropathy, but if they say yes, we will probably handle it differently.”* – Oncologist

Participants generally recognized that a clinician-dependent approach to symptom management and clinical consultations content meant that some issues went unaddressed. MyPath was seen as helpful for uncovering often overlooked problems, as explained by an allied HCP:

*“MyPath can give a more comprehensive picture of how the patient is truly doing... with current practices, it’s somewhat random whether issues are uncovered or not.”* – Allied health professional

Additionally, participants emphasized that having access to patient-reported data in advance, supported by symptom management guidelines, could help structure consultations around patient-identified concerns, making PCC more feasible in routine oncology practice. As one oncology nurse explained:

*“It would be beneficial to have [MyPath] integrated so we can address the points patients respond to, shaping the conversation around that.”* – Oncology nurse

However, participants frequently worried that a solution like MyPath could uncover more issues than what was possible to address given time and workload pressures. Participants consistently stressed the need for resources and organizational support during the implementation phase where work processes would need to be adapted and learned, as illustrated by an oncologist:

*“This will require resources...it’s not realistic to assume everything will improve without more staff and time to get it implemented.”* – Oncologist

### Customization needs and patient acceptance of digital tools

This theme reflects how successful adoption of MyPath depends on its adaptability to clinical needs and diversity, digital system compatibility, as well as patient acceptance to ensure usability across treatment modalities.

Most participants saw potential in technology for streamlining assessments, improving access to data, and simplifying documentation. However, many emphasized that digital tools needed to be adapted to the specific settings it was intended to be used. A one-size-fits-all approach was seen as problematic, as participants perceived that their patient group had unique concerns or symptoms, and that too many questions could be burdensome to review while too few could lack necessary specificity. Many participants suggested balancing general and disease-specific questions to ensure relevance:

*“Customizable questions would be beneficial...having both general and tumor-specific sections would enhance usability for different patients.”* – Oncologist

Participants also emphasized the need for integration with existing HIT systems to avoid duplicative work. Past experiences with digital solutions lacking interoperability were cited as adding burdens and limiting enthusiasm, a concern especially relevant for MyPath, which was not intended to replace existing systems:

*“We already manually connect information from different systems... if MyPath doesn’t integrate smoothly, but simply adds another program, it would be very impractical.”* – Oncologist

User-friendly design and intuitive interfaces were considered critical for sustained usage and initial enthusiasm. Many referenced past experiences with unintuitive digital solutions or digital tools that did not deliver what was promised, which generally seemed to make participants less enthusiastic about new digital tools, as illustrated by an oncologist:

*“I’m enthusiastic if it works well, but I’m reserved because of the challenges we’ve faced with digital tools before.”* – Oncologist

Additionally, concerns were raised about patients’ digital literacy, frailty, and willingness to engage with technology. Clinicians stressed the importance of support and feedback from patients and caregivers to ensure that tools added value without compromising care quality. One oncologist raised a concern regarding patient acceptance of digital follow-up:

*“It will be interesting to hear if patients prefer using forms and apps or value human contact more. I’m curious about their satisfaction.”* – Oncologist

### Combining patient-reported data with clinical autonomy

This theme describes clinicians’ perspectives on PROMs. While many saw potential in digital PROMs for efficiency and longitudinal tracking, and in making care more patient-centered, they emphasized that such tools should support rather than replace clinical judgment. Concerns included reliability of PROMs, the importance of contextual information, and the need for clinician involvement in tool development. Overall, participants valued digital PROMs for their potential in providing structure and historical overview, as illustrated by an allied health professional:

*“Having a historical overview saves you from searching through records. A systematic presentation would be very useful.”* – Allied health professional

However, participants cautioned against relying solely on PROMs, voicing concern that MyPath might be used primarily for remote monitoring rather than direct patient interaction, which they considered essential for PCC. They emphasized that PCC decisions are nuanced and require contextual, complementary, and interpersonal insights to fully understand and assess the holistic needs of patients. Including caregiver perspectives alongside PROMs was seen as important to increase their reliability, as illustrated by an oncology nurse:

*“Family members are very important...to have someone fill out the picture is quite important. Because sometimes you’re not sure if the patient has disclosed everything.” –* Oncology nurse

Additionally, some participants noted that the complexity of PCC and symptom management is difficult to capture digitally. Important information might be missed if PROMs are used in isolation rather than interactively with patients, which could reduce their usefulness in patient-centered assessments, as illustrated by an oncology nurse:

*“You need complete information... if there’s an indication for a treatment, but the patient can’t swallow tablets because they have a feeding tube...then it’s not very useful.” –* Oncology nurse

Finally, participants stressed the importance of clinician involvement in deciding PROM content and questions, workflow integration and digital design, seeing this as key to building confidence and ownership to the solution. As illustrated by an oncologist:

*“I think it is wise to get someone on board – some of the doctors in our group – who can actively support and promote it from within, so it does not just feel like an external initiative.”* – Oncologist

## Discussion

This study aimed to explore HCPs perceptions, expectations, and the perceived value of digitally supported PCC in diverse oncology outpatient clinics. Our findings highlight the tension between meeting the perceived needs of HCPs, such as alignment with existing routines and roles, and supporting HCPs to understand how digital PCC can be used to reshape their own clinical practice. While HCPs recognized the value of PCC and digital tools to assess patients’ needs, their perspectives were shaped by existing professional culture, practical challenges related to resources and workflow, and a desire to maintain clinical autonomy. The following discussion explores these findings in detail and outlines key considerations for the successful implementation of technology-supported PCC into oncology.

Participants recognized the value of PCC in principle but often viewed it as secondary to disease-centered approaches, preferring to focus on anticancer treatment over PCC. Notably, participants seemed to associate PCC more with palliative care, likely due to the overlapping focus on quality of life and symptom management. Similar descriptions and barriers have been identified in studies investigating the integration of palliative care in oncology, historically also faced with challenging integration into oncology [54, 55]. Despite modern oncology increasingly emphasizing long-term survivorship, symptom management, and patient experiences [8–12], oncology’s traditional focus on anticancer treatment contribute to perceptions that PCC falls outside its primary scope [55–57]. Overcoming these cultural and organizational barriers is critical to enabling systematic, integrated PCC, including comprehensive symptom assessment supported by PROMs and digital solutions.

The study also highlighted practical opportunities and challenges in implementing digital tools for PCC. Participants generally acknowledged the potential of MyPath to support structured symptom assessments, improve access to patient-reported data, and inform clinical decision-making. However, concerns were raised about increased workload, misalignment with existing workflows, and the risk that digital tools could undermine clinical autonomy. These perceptions reflect a broader challenge in adoption of digital health tools, where short-term resource demands and workflow adjustments may obscure potential long-term benefits [58–62]. These findings underscore the importance of stakeholder involvement in digital tool design and implementation. In particular, assessing necessary workflow adjustments to optimize digital tool integration into clinical practice will likely reduce initial skepticism, allowing HCPs to experience the potential benefits first-hand.

Many participants perceived a tension between standardization and personalization, worrying that standard processes might limit professional autonomy or individualized care, seen as essential aspects of PCC. They emphasized the importance of customizable content and PROMs to reflect individual patient needs in various contexts of cancer care. This is consistent with the recommendations by ESMO stating that PROMs serve different purposes across the cancer care continuum, helping to assess baseline health and frailty before treatment, monitor side effects during active therapy, and track long-term outcomes during follow-up [63]. Clearly communicating to clinicians that PROMs intend to be adapted to treatment intent and disease stage is essential to foster adoption and clinical usefulness [42, 65, 66].

Additionally, perceptions that digital tools and PROMs would replace, rather than enhance, patient consultations were common, with concerns that this would reduce clinical autonomy. These are common concerns regarding PROMs implementation [66–71], and underscore the need for training and instructions to HCPs that emphasize that PROMs are designed to support, not replace, clinical judgment. Training efforts should focus on moving beyond treating PROMs as fixed scores to determine treatment, but as a valuable vantage point for patient assessment and conversation to facilitate shared decision-making and PCC.

Consistent with prior research, HCPs’ in this study expressed concerns about patient frailty and low digital literacy reducing uptake of a digital solution. However, this may be more reflective of clinicians’ own uncertainties rather than actual patient reluctance, as studies on patient perspectives consistently demonstrate acceptance among patients [30, 32]. However, a recent systematic review identified that most studies on patient acceptance of digital tools use non-representative, convenience samples of individuals already using digital tools [72]. This raises the question whether studies on patient perspectives are transferable to real-world clinical oncology. To reinforce HCP confidence in patient acceptance and ensure the tool is usable across diverse patient groups, it is essential to involve representative, real-world patients in the development and testing of digital tools, providing HCPs with the confidence that patients are willing and able to engage with the solution as intended.

Lastly, participants focused predominantly on external barriers to digitally supported PCC such as limitations of digital tools and PROMs, and workforce and time disparities. Notably absent, however, were deeper reflections on the personal and professional adjustments needed to integrate digital PCC into routine clinical practice. This absence reinforces the broader tension identified throughout this study, between optimizing digital systems to fit existing workflows and enabling HCPs to evolve those workflows toward more PCC. This aligns with prior studies reporting that oncology HCPs often underrecognize the professional and personal changes required for successful and meaningful changes in clinical practice [55]. Addressing both technological and cultural factors through education, professional development, and organizational support is crucial to embedding PCC as a routine and valued part of oncology care, rather than something that competes with or contradicts tumor-directed therapies.

### Strengths and limitations

This study offers in-depth insights into diverse HCP perspectives; however, some limitations should be noted. The purposive sampling strategy guided by stakeholder analysis may have introduced bias, as PIs responsible for participant recruitment were oncologists and may have unintentionally overlooked other professional groups than nurses and doctors. Snowball sampling intended to mitigate this, however allied health professionals, who are recognized as vital in PCC, were not widely recruited in the study, highlighting an area for future research.

While thematic analysis facilitated systematic coding and theme development, it also presents limitations. Reflexive thematic analysis relies on continuous engagement and immersion with the data, allowing for the emergence of nuanced or unexpected themes [73]. However, data collection at different clinical sites necessitated the involvement of multiple interviewers and may have reduced the ability to fully build on emerging concepts during data collection. Additionally, while the use of a pre-specified framework (TPOM) provided an evidence-based approach to analysis it may have limited the identification of emergent themes. The research team’s established focus on palliative care, combined with conducting the study in parts at our own institution, may have unintentionally framed MyPath as more relevant to palliative care than intended, shaping participants’ perceptions and responses.

Lastly, this study explored HCP perspectives at a conceptual stage of MyPath. As the concept was further developed, participant validation meetings indicated that some perspectives had shifted to a more positive view, suggesting that opinions on MyPath and digitally enabled PCC are dynamic and may evolve over time. This highlights the value of follow-up studies at later stages to capture perspectives as the tool is implemented in real-world clinical settings.

## Conclusions

HCPs recognize the value of PCC in improving healthcare outcomes such as QoL and treatment satisfaction, and the potential of digital tools utilizing PROMs to support its integration in oncology. The findings highlight a tension between meeting HCPs’ perceived needs for technical functionality and workflow alignment and supporting them to meaningfully incorporate digital PCC into their clinical practice. Overcoming the cultural, organizational, and professional barriers that currently undermine PCC will require strong stakeholder engagement and effective change management. Repositioning PCC as an integral rather than competing part of high-quality cancer care is essential to achieving lasting implementation.

## Supplementary Information

Below is the link to the electronic supplementary material.

Supplementary Material 1.

## Data Availability

The datasets generated and/or analyzed during the current study are not publicly available due to the sensitive and identifiable nature of qualitative interview data. Participants were ensured that their confidentiality would be protected, and public sharing of the transcripts would compromise their anonymity. Reasonable requests for limited access to anonymized excerpts may be considered by the corresponding author, subject to ethical and participant consent.

## Abbreviations

PCC: Patient-centered care
QoL: Quality of life
TCC: Tumor-centered care
PROMs: Patient-reported outcome measures
HCPs: Healthcare professionals
HIT: Health information technology
EU: European union
COREQ: Consolidated criteria for reporting qualitative research
PIs: Principal investigators
TPOM: Technology, people, organizations, and macroenvironmental factors
DPOs: Data protection officers
NVivo: Software for qualitative data analysis (QSR International)
